# Endobronchial Ulceration: A Different Manifestation of Antineutrophil Cytoplasmic Antibodies (ANCA)-Associated Vasculitis

**DOI:** 10.7759/cureus.62596

**Published:** 2024-06-18

**Authors:** Ana Sara Gonçalves, Filipa Menezes Freitas, Cláudia Sousa, João Carvalho, Vítor Teixeira

**Affiliations:** 1 Pulmonology Department, Hospital Central do Funchal, Funchal, PRT

**Keywords:** granulomatosis with polyangiitis, staphylococcus aureus, vasculitis, ulceration, bronchoscopy

## Abstract

Vasculitis with pulmonary involvement is often associated with antineutrophil cytoplasmic antibody (ANCA). We describe a case involving a 66-year-old male patient diagnosed with granulomatosis with polyangiitis (GPA). Following the onset of hemoptysis, bronchoscopy revealed endobronchial ulcers correlated with GPA. Bronchial biopsies and bronchoalveolar lavage identified *Staphylococcus aureus* (*S. aureus*), which is associated with GPA relapses. The patient began immunosuppression to induce remission, and Avacopan was subsequently introduced for the treatment of GPA and microscopic polyangiitis (MPA).

## Introduction

Granulomatosis with polyangiitis (GPA) is characterized by necrotizing granulomatous inflammation and vasculitis that involves the upper and lower respiratory tracts, as well as kidneys, skin, eyes, and joints [1]. The first case was described by Heinz Klinger in 1931 [1], and it is estimated to affect 12 to 14 individuals per million worldwide [2]. The age of diagnosis is usually between 40 and 65 years old, affecting males and females equally [2].

Various pulmonary manifestations associated with this disease are described in the literature, namely lung nodules with or without cavitation [3], infiltrates or alveolar opacities that can be bilateral, diffuse, or localized (consolidation), and endobronchial alterations such as bronchial stenosis, ulcerations, or pseudotumors [4].

## Case presentation

We describe a case of a 66-year-old male patient, a non-smoker with a history of arterial hypertension, type 2 diabetes mellitus, coxarthrosis, and chronic alcohol consumption. He worked as a truck driver and had no relevant environmental exposure. He presented at the ED with hemoptysis, odynophagia, dyspnea, and a productive cough that had been ongoing for two weeks. Additionally, he experienced polyarthralgia with an inflammatory rhythm, as well as oral and cutaneous ulcers. He also reported significant weight loss (20 kg in one year) and night sweats. Upon examination at the ED, pulmonary auscultation revealed bilateral crackles, arterial blood gas indicated new onset type 1 respiratory failure, and blood analysis showed normocytic/normochromic anemia, a creatinine level of 1.59 mg/dL, and a C-reactive protein level of 202 mg/L (Table 1).

**Table 1:** Blood test results.

Parameter	Result	Reference range
White cell count (x10^3^/mL)	5.15	4.2-10.8
Neutrophils (x10^3^/mL)	3.5	1.9-7.2
Lymphocytes (x10^3^/mL)	1.0	1.2-3.4
Monocytes (x10^3^/mL)	0.2	0.3-0.9
Eosinophils (x10^3^/mL)	0.4	0.0-0.6
Basophils (x10^3^/mL)	0.0	0.0-0.1
Erythrocytes (x10^6^/mL)	2.53	4.37-5.74
Hemoglobin (g/dL)	8.0	13.7-17.3
Hematocrit (%)	25.2	40.0-51.0
Mean corpuscular volume (fL)	96.5	80.0-99.0
Red cell distribution width (%)	12.9	11.5-15.0
Platelets (x10^3^/mL)	285.0	144.0-440.0
Glucose (mg/dL)	143.0	82.0-115.0
Urea (mg/dL)	54.0	16.6-48.5
Creatinine (mg/dL)	1.59	0.70-1.20
Estimated glomerular filtration rate (eGFR) (ml/min/1.73 m^2^)	48	
Sodium (mEq/L)	138	135-145
Potassium (mEq/L)	3.90	3.50-5.10
Chloride (mEq/L)	100	98-107
Aspartate aminotransferase (AST) (U/L)	52.0	≤​40.0
Alanine aminotransferase (ALT) (U/L)	28.1	≤41.0
Lactate dehydrogenase (LDH) (U/L)	261.0	<250.0
Creatine kinase (CK) (U/L)	56.0	<190.0
C-reactive protein (mg/L)	202.0	<5.00

**Figure 1:**
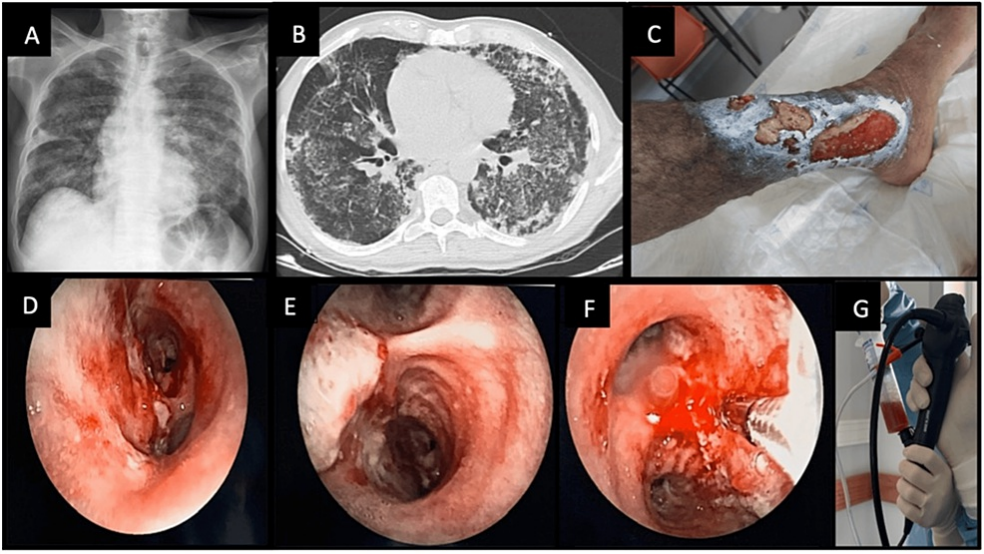
Clinical case illustration. A, B: X-ray and thoracic CT scan showing bilateral diffuse cotton-wool infiltrate and small emphysematous bullae. C: Leg cutaneous ulcer. D, E, F: Ulcerated lesions/white plaques in the carina, progressing to the right main bronchus. G: Bronchoalveolar lavage (BAL) performed in the middle lobe, with increasingly hemorrhagic fluid return.

A thoracic X-ray displayed bilateral diffuse infiltrate (Figure 1A), and a thoracic CT scan revealed extensive bilateral diffuse cotton-wool infiltrate, associated with tree-in-bud images suggestive of an inflammatory or infectious process, and small emphysematous bullae at the lung apex (Figure 1B). The patient was admitted to the pulmonology department for treatment and further investigation of his condition. Cultures from a leg ulcer detected the presence of methicillin-resistant Staphylococcus aureus (MRSA) and Pseudomonas aeruginosa (Figure 1C). A bronchoscopy showed ulcerated lesions and white plaques in the carina, extending to the right main bronchus (Figures 1D-1F). Bronchial biopsies taken from the right main bronchus revealed fragments of bronchial mucosa with abscess formation in the chorion, areas of necrosis, and metaplasia, without signs of dysplasia or malignant neoplasms. Bronchoalveolar lavage (BAL) and brushings from the middle lobe yielded increasingly hemorrhagic fluid (Figure 1G), and cultures confirmed the presence of MRSA, which was also found in bronchial biopsies and a sputum sample. Nasal exudate, blood cultures, and serologies were negative, as was the polymerase chain reaction (PCR) test for Mycobacterium tuberculosis. The alpha-1 antitrypsin value was within the normal range.

CT pulmonary angiogram excluded pulmonary embolism, and echocardiogram did not reveal vegetations on the valves. An autoimmune study detected the presence of anti-proteinase 3 (PR3) antibody, 205.9 U/mL, a type of anti-neutrophil cytoplasmic antibody (c-ANCA) [1]. A 24-hour urine collection uncovered 1700 mg of protein and erythrocyturia. Consequently, the patient underwent a kidney biopsy, which diagnosed crescent glomerulonephritis with fibrosis and tubular atrophy (<25%) and scarce IgM deposits. Indeed, GPA associated with PR3-ANCA, presenting with pulmonary, renal, mucocutaneous, and hematological involvement, was our final diagnosis. Induction of remission began with three pulses of methylprednisolone 1g, followed by oral prednisone 1 mg/kg per day and two doses of rituximab 1 g, with an interval of 14 days between doses. The patient maintained follow-up in rheumatology, pulmonology, nephrology, and physical and rehabilitation medicine consultations. Avacopan was subsequently started with a good response, facilitating corticosteroid weaning.

## Discussion

Vasculitis represents a heterogeneous group of pathologies with varied clinical expressions. Respiratory manifestations are frequent in ANCA-associated vasculitis. Endobronchial ulceration, without bronchial stenoses, is an interesting pattern observed in this patient and is an incidental finding that hallmarks GPA in this case.

Clinical evidence shows that nasal colonization with *S. aureus* is a risk factor for GPA relapse [5]. Although the patient we present does not have a positive nasal exudate for this microorganism, MRSA was detected in a sputum sample, bronchoalveolar lavage, microbiology of bronchial biopsies, and leg ulcer culture, possibly linked to the exacerbation of his disease.

Treatment of GPA should be based on the latest international recommendations, allowing better results and lower associated toxicity. The patient started an induction regimen with glucocorticoids in combination with rituximab. Avacopan was initiated after induction of remission with a good response, enabling corticosteroid weaning.

Avacopan is an orally administered therapy for GPA or microscopic polyangiitis (MPA) that blocks the complement C5a receptor (C5aR) and stops the activation of neutrophils, thereby reducing inflammation of blood vessels [6]. It represents a new class of medication that helps to control the disease and permits extended remission of this pathology. This patient started Avacopan and remains stable, with no new relapses.

## Conclusions

Accurate diagnosis of GPA is crucial due to the high morbidity and mortality in affected individuals. Diagnosing GPA can be challenging because of its clinical similarities to other systemic rheumatic diseases.

The most common pulmonary symptoms of vasculitis with pulmonary involvement are hemoptysis and dyspnea. Indeed, pulmonary alterations seen in exams such as X-rays or CT scans may be associated with lung diseases or systemic pathologies. This case underscores the importance of thorough investigation in patients presenting with multiple signs and symptoms and illustrates the significance of collaboration between pulmonology and rheumatology.

## References

[1] Garlapati P, Qurie A (2024). Granulomatosis with polyangiitis. https://www.ncbi.nlm.nih.gov/books/NBK557827/..

[2] (2024). Vasculitis Foundation: About Granulomatosis With Polyangiitis. https://www.vasculitisfoundation.org/education/vasculitis-types/granulomatosis-with-polyangiitis/.

[3] Gómez-Gómez A, Martínez-Martínez M, Cuevas-Orta E (2014). Manifestaciones pulmonares de la poliangeítis granulomatosa. Reumatol Clin.

[4] Cordier JF, Valeyre D, Guillevin L, Loire R, Brechot JM (1990). Pulmonary Wegener's granulomatosis. A clinical and imaging study of 77 cases. Chest.

[5] Richter AG, Stockley RA, Harper L, Thickett DR (2009). Pulmonary infection in Wegener granulomatosis and idiopathic pulmonary fibrosis. Thorax.

[6] Harigai M, Takada H (2022). Avacopan, a selective C5a receptor antagonist, for anti-neutrophil cytoplasmic antibody-associated vasculitis. Mod Rheumatol.

